# Broad-Spectrum Regulation of Nonreceptor Tyrosine Kinases by the Bacterial ADP-Ribosyltransferase EspJ

**DOI:** 10.1128/mBio.00170-18

**Published:** 2018-04-10

**Authors:** Dominic J. Pollard, Cedric N. Berger, Ernest C. So, Lu Yu, Kate Hadavizadeh, Patricia Jennings, Edward W. Tate, Jyoti S. Choudhary, Gad Frankel

**Affiliations:** a Centre for Molecular Microbiology and Infection, Department of Life Sciences, Imperial College, London United Kingdom, London, United Kingdom; b Department of Chemistry, Imperial College, London United Kingdom, London, United Kingdom; c Functional Proteomics Group, Chester Beatty Laboratories, Institute of Cancer Research, London, United Kingdom; d University of California at San Diego, La Jolla, California, USA; GSK Vaccines

**Keywords:** ADP-ribosyltransferase toxins, *Citrobacter rodentium*, infection, T3SS

## Abstract

Tyrosine phosphorylation is key for signal transduction from exogenous stimuli, including the defense against pathogens. Conversely, pathogens can subvert protein phosphorylation to control host immune responses and facilitate invasion and dissemination. The bacterial effectors EspJ and SeoC are injected into host cells through a type III secretion system by enteropathogenic and enterohemorrhagic Escherichia coli (EPEC and EHEC, respectively), Citrobacter rodentium, and Salmonella enterica, where they inhibit Src kinase by coupled amidation and ADP-ribosylation. C. rodentium, which is used to model EPEC and EHEC infections in humans, is a mouse pathogen triggering colonic crypt hyperplasia (CCH) and colitis. Enumeration of bacterial shedding and CCH confirmed that EspJ affects neither tolerance nor resistance to infection. However, comparison of the proteomes of intestinal epithelial cells isolated from mice infected with wild-type C. rodentium or C. rodentium encoding catalytically inactive EspJ revealed that EspJ-induced ADP-ribosylation regulates multiple nonreceptor tyrosine kinases *in vivo*. Investigation of the substrate repertoire of EspJ revealed that in HeLa and A549 cells, Src and Csk were significantly targeted; in polarized Caco2 cells, EspJ targeted Src and Csk and the Src family kinase (SFK) Yes1, while in differentiated Thp1 cells, EspJ modified Csk, the SFKs Hck and Lyn, the Tec family kinases Tec and Btk, and the adapter tyrosine kinase Syk. Furthermore, Abl (HeLa and Caco2) and Lyn (Caco2) were enriched specifically in the EspJ-containing samples. Biochemical assays revealed that EspJ, the only bacterial ADP-ribosyltransferase that targets mammalian kinases, controls immune responses and the Src/Csk signaling axis.

## IMPORTANCE

Enteropathogenic and enterohemorrhagic Escherichia coli (EPEC and EHEC, respectively) strains cause significant mortality and morbidity worldwide. Citrobacter rodentium is a mouse pathogen used to model EPEC and EHEC pathogenesis *in vivo*. Diarrheal disease is triggered following injection of bacterial effectors, via a type III secretion system (T3SS), into intestinal epithelial cells (IECs). While insights into the role of the effectors were historically obtained from pathological, immunologic, or cell culture phenotypes, subtle roles of individual effectors *in vivo* are often masked. The aim of this study was to elucidate the role and specificity of the ADP-ribosyltransferase effector EspJ. For the first time, we show that the *in vivo* processes affected by a T3SS effector can be studied by comparing the proteomes of IECs extracted from mice infected with wild-type C. rodentium or an *espJ* catalytic mutant. We show that EspJ, the only bacterial ADP-ribosyltransferase that targets mammalian kinases, regulates the host immune response *in vivo*.

## INTRODUCTION

Protein phosphorylation has been documented on 75% of the human proteome, with the number of human phosphorylation sites estimated to be several hundred thousand (1). The human genome encodes 58 membrane-spanning receptor tyrosine kinases (RTKs) and 32 nonreceptor tyrosine kinases (NRTKs), which are reported to be responsible for <1% of the total protein phosphorylation (2). Nonetheless, tyrosine phosphorylation coordinates the response to a multitude of intracellular and extracellular triggers, including growth factor signaling, cell cycle control, regulation of transcription factors, and the transduction of signaling from immune cell receptors.

The NRTKs include a range of regulatory domains accompanying the core kinase domain. The Src family kinases (SFKs) include eight NRTKs, i.e., the ubiquitous Src, Yes1, and Fyn proteins and the classically hematopoietic Hck, Lck, Lyn, Fgr, and Blk proteins. SFKs contain N-terminal lipidation sites for membrane tethering, followed by Src homology 3 (SH3) and SH2 domains and an SH1 kinase fold. The SH3 and SH2 domains provide autoregulation and substrate specificity through their respective proline-rich region and phosphotyrosine-binding capacities (3). This SH3-SH2-SH1 core is conserved in the C-terminal Src kinase (Csk), Abl, and Tec families (4). Csk is recruited to sites of SFK activity, where it inhibits the SFKs through phosphorylation of their unique C-terminal tyrosine (5). Abl kinase contains additional DNA- and actin-binding domains and modulates the cell cycle, morphology, and motility. Tec kinases have a pleckstrin homology domain for membrane tethering, where they signal downstream of antigenic B- and T-cell receptors stimulating phospholipase Cγ and calcium mobilization and regulating actin reorganization and cell polarization (6). In Syk kinases, the SH3 domain is replaced with a second SH2 domain enabling their recruitment to phosphorylated immunoreceptor tyrosine activation motifs of activated B-cell, T-cell, and Fcγ receptors (FcγR) (7). Crucially, the Abl, Tec, and Syk kinases may each be regulated by SFKs, which explains the plethora of overlapping roles attributed to the SFKs. Misregulation of tyrosine kinases has been associated with many diseases, including cancer. Accordingly, these proteins have long been attractive therapeutic targets (8). As tyrosine phosphorylation is also central to the host response to infection, it is often targeted by pathogens to evade the immune system. Moreover, the infection strategy of many viral and bacterial pathogens involves the exploitation of host protein kinases for multiplication and dissemination.

The enteric human pathogens enteropathogenic Escherichia coli (EPEC) and enterohemorrhagic E. coli (EHEC) and the mouse pathogen Citrobacter rodentium are known as attaching and effacing (A/E) pathogens. Infection with C. rodentium causes colitis and colonic crypt hyperplasia (CCH) (9). Colonization of the gastrointestinal tract by these pathogens is meditated by a type III secretion system (T3SS), which injects bacterial effector proteins into the host cell cytosol to hijack signaling for the benefit of the bacterium. Numerous T3SS effectors have an enzymatic activity, including the glycosyltransferase NleB, the methyltransferase NleE, and metalloproteases NleC and NleD, which can inhibit host inflammatory pathways (10). A characteristic feature of infection by A/E pathogens is the formation of actin-rich pedestals following clustering of the effector Tir in the plasma membrane by the bacterial adhesin intimin; pedestal formation by EPEC and C. rodentium is dependent on Tir tyrosine phosphorylation by a range of redundant NRTKs, including members of the Abl/Arg and Src families (11).

Recently, we have shown that the effector EspJ is an ADP-ribosyltransferase (ART) that inhibits complement receptor 3 (CR3)- and FcγR-mediated phagocytosis via the inhibitory ADP-ribosylation of Src (12). Moreover, we have shown that the EspJ homologues SeoC and SboC, expressed by Salmonella enterica subsp. salamae and arizonae and S. bongori, respectively, also ADP-ribosylate the highly conserved Src glutamic acid residue at position 310 (E310) to inhibit phagocytosis (13). EspJ is a novel ART, as it simultaneously amidates and ADP-ribosylates Src E310, resulting in an ADP-ribose-conjugated glutamine. This study aimed to determine the repertoire of EspJ substrates and its role during *in vivo* infection.

## RESULTS

### EspJ regulates the host immune response *in vivo.*

Previous studies of EspJ were performed mainly by infecting cultured cells for a few hours (12, 13). However, *in vivo*, A/E pathogens primarily interact with intestinal epithelial cells (IECs) over several days or weeks. Indeed, by quantifying proteomic responses of IECs isolated from mice 8 days postinfection with wild-type (WT) C. rodentium, we have recently shown that it affects cellular bioenergetics and cholesterol homeostasis (14).

**FIG 1:**
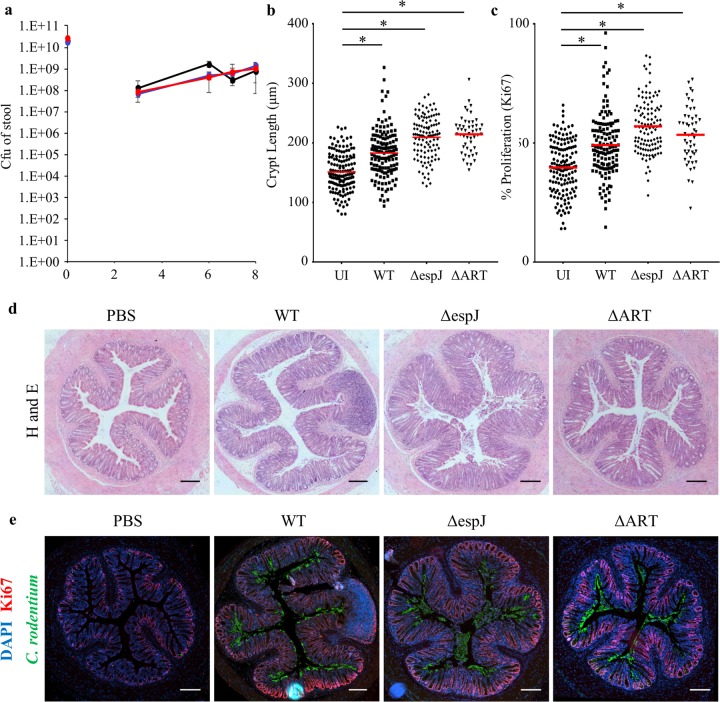
C. rodentium Δ*ART* colonizes mice and induces CCH. C57BL/6 mice were inoculated with WT C. rodentium, C. rodentium Δ*espJ*, an EspJ catalytic mutant (Δ*ART*), or PBS. CFU, colony forming units. (a) Colonization was assessed by determining the number of CFU per gram of stool sample at days 3, 6, 7, and 8 postinfection. Δ*ART* mutant (red), Δ*espJ* mutant (blue), and WT (black) C. rodentium were shed equally. Crypt lengths (b) and proliferation (Ki-67 staining) (c) were measured to assess CCH in H&E-stained sections of the distal colon from PBS- or C. rodentium (WT, Δ*espJ*, or Δ*ART*)-injected mice. The Δ*ART* mutant and WT strains significantly induced lengthening of the crypts to similar levels. UI, uninfected. (d, e) Representative H&E-stained and immunofluorescence section images (4',6-diamidino-2-phenylindole [DAPI], blue; Ki-67, red; C. rodentium, green). Statistical significance was determined by one-way analysis of variance, followed by Bonferroni’s post-test. *, *P* < 0.05. Red line, mean crypt length. Bars in panels d and e, 200 μm.

We first analyzed the infection dynamics after oral gavage of mice with WT or Δ*espJ* or Δ*ART* (Δ*espJ* mutant complemented on the genome with *espJ*_R79A_ catalytic mutation) mutant C. rodentium. Each strain showed similar colonization dynamics (Fig. 1a) and induced comparable levels of CCH (Fig. 1b to e), as measured by colonic crypt length and Ki-67 staining (a marker for cell proliferation). To investigate the role of EspJ *in vivo*, we then performed proteomics analysis of IECs purified from mice at the peak of shedding, 8 days postchallenge with WT or Δ*ART* mutant C. rodentium. We used isobaric peptide labeling (tandem mass tag [TMT]) and MS3 quantification, as recently reported (14), to determine protein abundance changes in IECs isolated from mock-infected mice or mice infected with WT or Δ*ART* C. rodentium (four, five, and six mice per group, respectively).

**FIG 2:**
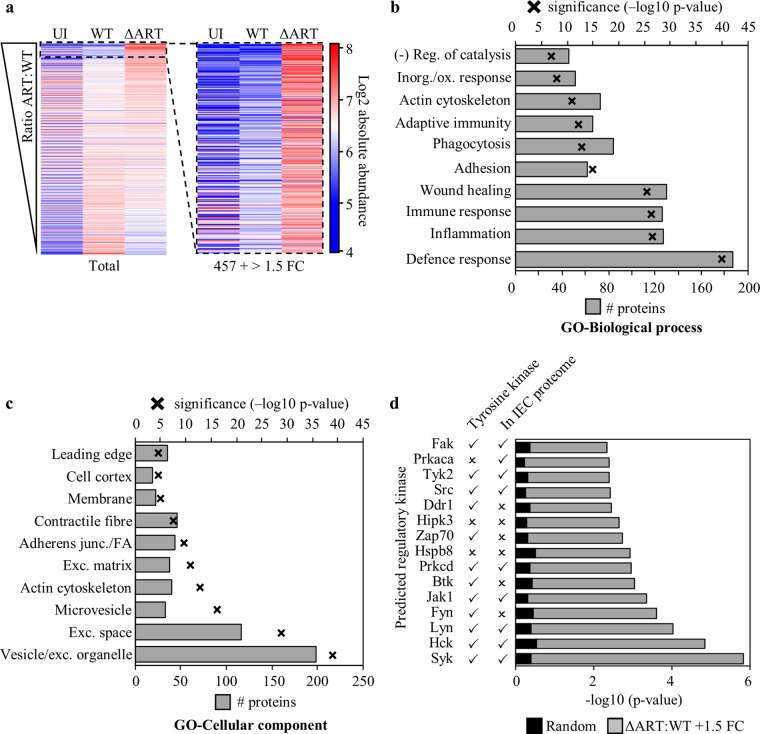
EspJ is an immunosuppressant that regulates multiple tyrosine kinases *in vivo*. Proteomes of IECs purified from mice inoculated with WT C. rodentium, a C. rodentium EspJ catalytic mutant (Δ*ART*), or PBS (uninfected [UI]). (a) A ratio of Δ*ART* mutant to WT protein abundances was created and ranked according to those that increased the most in Δ*ART* mutant- versus WT-infected IECs (left). The enlarged image shows 457 proteins with abundance increases of at least 1.5-fold in Δ*ART* mutant compared to WT infections; which were assigned to 10 GO term groups for biological process (b) and cellular components (c). Analyses are shown with the number of GO term-associated proteins in grey bars and the negative log *P* value (with Bonferroni correction) indicated by black crosses. Abbreviations: (−) Reg., negative regulation; Inorg./ox. response, response to inorganic substance/oxidative stress; Exc., extracellular; FA, focal adhesion. (d) The 15 most likely upstream regulatory kinases targeted by EspJ to produce the observed changes in protein abundance. Grey bars show the kinase *P* value for the group of EspJ-modulated proteins, and black bars show the mean *P* value of the kinase across X2K runs from 10 random lists of 457 proteins from the total of 7,400. Check marks (✓) highlight the tyrosine kinases within this prediction and whether the kinase was identified in the IEC proteome. See also Fig. S1 and S2.

We identified 7,400 mouse proteins and quantified them by shotgun proteomics analysis of isobarically labeled IEC proteins (false-discovery rate [FDR]-corrected *P* value, <0.01). Compared to the protein abundances in uninfected IECs, those of 3,393 protein abundances were changed by ±1.5-fold upon WT infection and those of 3,164 were changed upon Δ*ART* mutant infection, with 2,650 of these changes observed in both infections (see Fig. S1a in the supplemental material). We performed KEGG pathway gene ontology (GO) term analysis of the proteins with ±1.5-fold abundance changes upon WT or Δ*ART* infection. The pathways regulated by WT and Δ*ART* infections were very similar and reflected observations in our previous report (14) (Fig. S1b and c). In comparison to WT infection, 457 proteins were increased >1.5-fold in the absence of active EspJ (Δ*ART* infection), suggesting that these proteins are induced during WT infection but directly or indirectly suppressed by EspJ catalysis (Fig. 2a; Table S3).

10.1128/mBio.00170-18.2 FIG S1  Results of proteomic analyses of IECs from uninfected and C. rodentium (WT or Δ*ART* mutant)-infected mice. Proteomes of IECs from mice inoculated with WT C. rodentium, a C. rodentium EspJ catalytic mutant (Δ*ART*), or PBS (uninfected, UI) are shown. (A) Venn diagram showing that of the total of 7,400 proteins identified (white), the abundances of 3,393 and 3,164 changed by ±1.5-fold when WT (dark gray) and Δ*ART* mutant (light gray) C. rodentium-infected and uninfected IECs, respectively, were compared. A total of 2,650 of these changes were common to WT and Δ*ART* mutant infections. (B, C) KEGG pathway analysis of these 3,393 (WT versus uninfected) and 3,164 (Δ*ART* mutant versus uninfected) proteins. Black crosses show the negative log_10_ Bonferroni-corrected *P* value for each predicted pathway, and bars represent the percentage of that pathway present in the group of proteins upregulated (gray bars) or downregulated (black bars) by WT (B) or Δ*ART* mutant (C) infection. Download FIG S1, PDF file, 0.6 MB. Copyright © 2018 Pollard et al. 2018 Pollard et al. This content is distributed under the terms of the Creative Commons Attribution 4.0 International license.

10.1128/mBio.00170-18.4 TABLE S1  Primers used to generate plasmids and strains new to this study, including restriction enzymes used for cloning. Download TABLE S1, DOCX file, 0.01 MB. Copyright © 2018 Pollard et al. 2018 Pollard et al. This content is distributed under the terms of the Creative Commons Attribution 4.0 International license.

10.1128/mBio.00170-18.5 TABLE S2  Plasmids used in this study and their sources. Download TABLE S2, DOCX file, 0.02 MB. Copyright © 2018 Pollard et al. 2018 Pollard et al. This content is distributed under the terms of the Creative Commons Attribution 4.0 International license.

10.1128/mBio.00170-18.6 TABLE S3  IEC proteomes. Shown is a spreadsheet with details of the proteomes from uninfected and WT- or Δ*ART* mutant-infected mouse IECs including the 7,400 identified proteins and groups of proteins with abundance changes of greater than ±1.5-fold in comparisons of WT-infected and uninfected, Δ*ART* mutant-infected and uninfected, or Δ*ART* mutant- and WT-infected IECs. In addition, all identified kinases or tyrosine kinase only are sorted into groups. Download TABLE S3, XLSX file, 1.7 MB. Copyright © 2018 Pollard et al. 2018 Pollard et al. This content is distributed under the terms of the Creative Commons Attribution 4.0 International license.

ClueGO biological process analysis of these 457 proteins suggests that EspJ has immunoregulatory roles, modulating proteins linked to the defense response to other organisms, regulation of immune cell migration and adhesion, cytokine responses, phagocytosis, and manipulation of the actin cytoskeleton (Fig. 2b). ClueGO cell component analysis revealed that these proteins are localized in and around the cell periphery; vesicles/phagosome, membrane, cytoskeleton/cortex, cell adhesions, and extracellular matrix (Fig. 2c). This is consistent with the fact that Src, the known EspJ target, is membrane tethered and has significant roles in the innate immune response and the actin cytoskeleton (15). However, considering that EspJ ADP-ribosylates a universally conserved kinase domain residue (Src E310) (12) and its broad effect during infection (Fig. 2), we hypothesized that its catalytic activity may impact multiple kinases.

### EspJ regulates numerous tyrosine kinases *in vivo.*

We used the kinase enrichment analysis (KEA) function within the Expression2Kinases (X2K) software to predict upstream regulatory kinases from the group of 457 proteins repressed by EspJ (16). KEA draws from kinase perturbation studies within numerous protein and gene expression databases to enable the prediction of upstream regulatory kinases responsible for changes in protein abundances. Twelve of the 15 most probable kinases regulated by EspJ were tyrosine kinases; 10 of these are NRTKs (Fig. 2d), of which 7 were present in the IEC proteome. Importantly, none of these kinases were significantly predicted from 10 randomly generated sets of 457 proteins (Fig. 2d; Table S4). When Δ*ART* mutant-infected and WT-infected IECs were compared, the abundance of Hck increased by >2-fold, but it was the only kinase from this group to change by >1.5-fold (Table 1). In conclusion, the *in vivo* proteomics data are consistent with the hypothesis that EspJ regulates multiple tyrosine kinases, including members of the Src, Fak, Jak, and Syk NRTK subfamilies (Fig. 2d; Table S4).

10.1128/mBio.00170-18.7 TABLE S4  X2K result spreadsheet. Shown is a spreadsheet with the results of X2K analysis of the 457 proteins upregulated in IECs when Δ*ART* mutant and WT infections are compared, as well as results from the analysis of 10 groups of 457 random proteins from the mouse IEC proteome. This includes the *P* value, *Z* score, and combined scores for each predicted kinase. Download TABLE S4, XLSX file, 0.2 MB. Copyright © 2018 Pollard et al. 2018 Pollard et al. This content is distributed under the terms of the Creative Commons Attribution 4.0 International license.

**TABLE 1:**
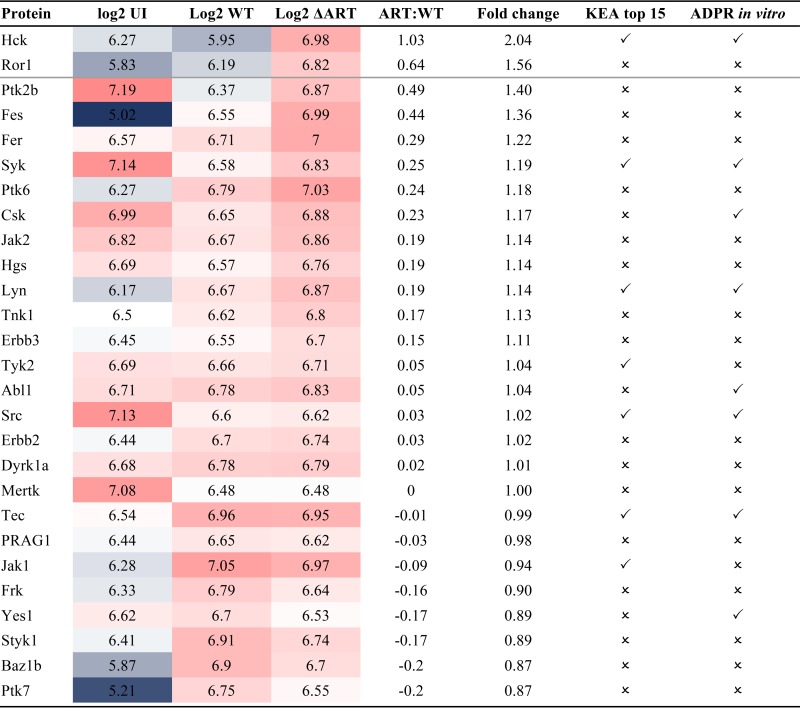
Kinases identified in IEC proteomes^a^

a Shown are the kinases identified in the proteomes of uninfected IECs and IECs infected with WT or Δ*ART* mutant C. rodentium, their respective log_2_ abundances, and the fold differences between Δ*ART* mutant and WT infection conditions. In addition, whether they were predicted to be regulated by EspJ *in vivo* by KEA or ADP-ribosylated by EspJ in cell lysate is noted. Protein log_2_ abundances range from blue (low) to white (medium) to red (high). UI, uninfected.

### EspJ can ADP-ribosylate a suite of NRTKs.

To test the hypothesis that EspJ regulates the activity of multiple kinases, we investigated its substrate repertoire in numerous cell lysates. Recombinant EspJ_EHEC_ and 2-ethynyl-adenosine-NAD (eNAD) were incubated with cell lysates before the conjugation of biotin onto resulting ADP-ribosylated proteins (17) and their enrichment with NeutrAvidin resin. Proteins were quantified by label-free quantitation (LFQ) mass spectrometry (MS) (PRIDE project PXD008533). Proteins enriched by the presence of EspJ were identified by using a two-sided *t* test and with an FDR-corrected *P* value of <0.01 and a fudge factor (*s*_0_) value of 1.

**FIG 3:**
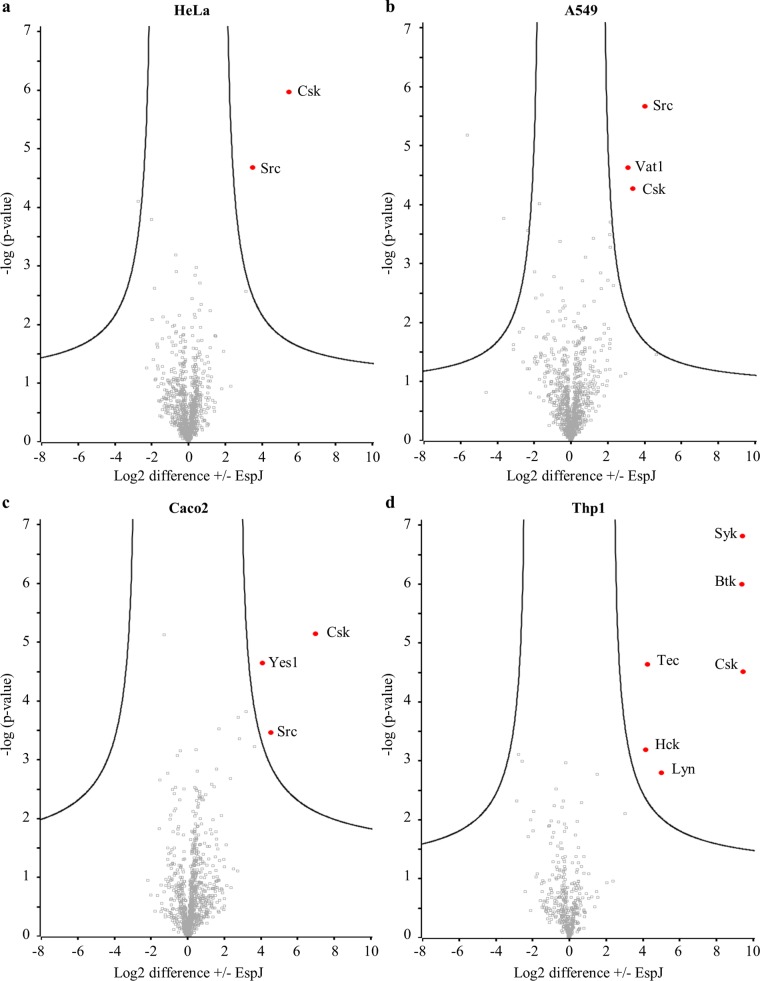
*In vitro* chemical proteomics analysis suggests that EspJ can ADP-ribosylate multiple tyrosine kinases. Lysates from HeLa (a), A549 (b), polarized Caco2 (c), and differentiated Thp1 (d) cells were incubated with EspJ_EHEC_ and eNAD. ADP-ribosylated proteins were tagged with biotin by click chemistry before enrichment with NeutrAvidin resin and analysis by LFQ MS. Red dots represent proteins that were significantly enriched when abundances in the presence versus the absence of EspJ were compared. Data sets were subjected to a two-sided *t* test, and black lines display the significance cutoff with an FDR-corrected *P* value of <0.01 and an *s*_0_ value of 1. See also Fig. S2.

**FIG 4:**
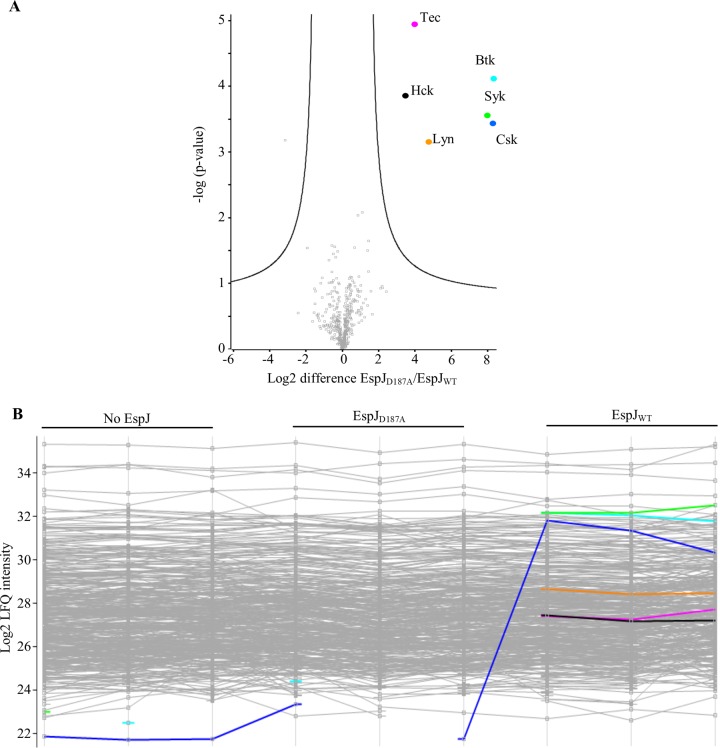
EspJ ADP-ribosylation of NRTKs is dependent on a functional Δ*ART* domain. Lysate from differentiated Thp1 cells was incubated with EspJ_EHEC_ (WT or D187A mutant) and eNAD. ADP-ribosylated proteins were tagged with biotin by click chemistry before enrichment with NeutrAvidin resin and analysis by LFQ MS. (A) Volcano plot after two-sided *t* test with a significance cutoff of an FDR-corrected *P* value of <0.01 and an *s*_0_ value of 1. (B) Profile plot of individual sample LFQ intensities. Blue, Csk; black, Hck; orange, Lyn; light blue, Btk; magenta, Tec; green, Syk.

**FIG 5:**
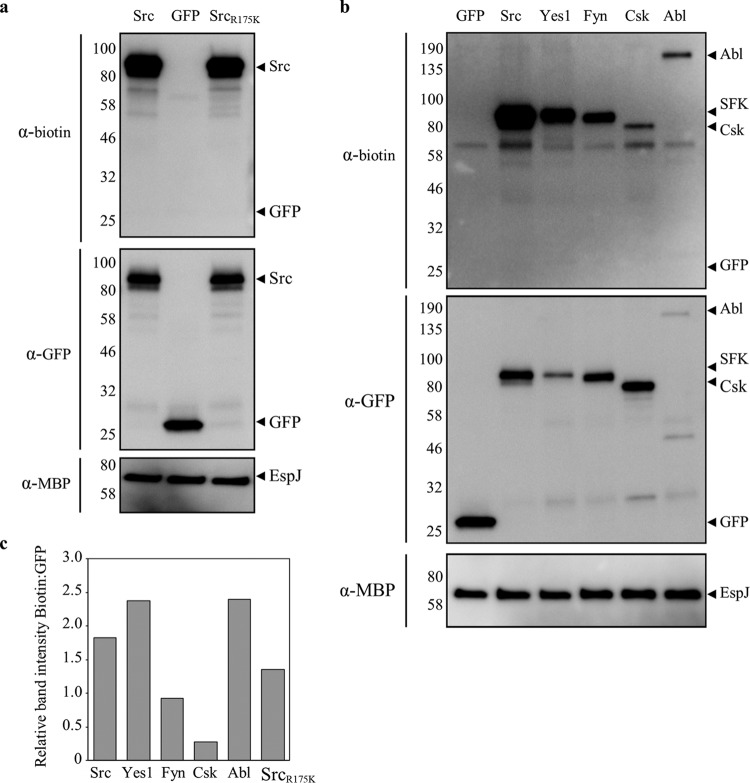
EspJ ADP-ribosylation of NRTKs displays specificity. HeLa cells were transfected with GFP, GFP-tagged Src, or Src_R175K_ (Src_RK_, SH2 domain mutant) (a) and Src, Yes1, Fyn, Csk, and Abl (b). The immunoprecipitated NRTKs were incubated with MBP-EspJ_EHEC_ and NAD-biotin. ADP-ribosylated proteins were detected by Western blotting and anti-biotin reagents. (c) The ratio of band intensities for ADP-ribosylated (biotin) to total (GFP) fluorescence was assessed by densitometry, and the mean ratio of three to five repeats is displayed for each transfected protein. EspJ has a preference for Src, Yes1, and Abl over Fyn and Csk, and the Src SH2 domain mutant (RK) has no impact on ADP-ribosylation by EspJ. The values to the left of the gels are molecular sizes in kilodaltons.

In HeLa (human cervical epithelial) and A549 (human alveolar epithelial) cell lysates, Src and Csk were significantly enriched (Fig. 3a and b). In polarized Caco2 (human epithelial colorectal) cell lysate, Src and Csk were again enriched, along with the SFK Yes1 (Fig. 3c). In differentiated Thp1 cells (human monocytes), lysate EspJ modified the largest range of targets, i.e., Csk, the SFKs Hck and Lyn, the Tec family kinases Tec and Btk, and the adapter tyrosine kinase Syk. Failure to enrich any of these kinases after incubation with the inactive maltose binding protein (MBP)-EspJ_D187A_ showed that their modification depends upon a functional EspJ ART domain (Fig. 4). Closer analysis reveals that Abl (HeLa and Caco2 lysates) and Lyn (Caco2 lysate) are enriched specifically in the EspJ-containing samples but below the significance cut-off after imputation for missing values (Fig. S2 and Table S5). This shows that EspJ can ADP-ribosylate a range of NRTKs from multiple cell lineages but suggests specificity within the tyrosine kinases due to the absence of other key NRTKs. Further, despite the proteomic identification of RTKs and serine/threonine kinases in these cell lines, none were confidently enriched (Table S5). The SH2 domain is common to the Src, Tec, Syk, and Csk subfamilies but is not found in the FAK and JAK subfamilies. To test whether SH2 plays a role in kinase recognition, green fluorescent protein (GFP)-tagged Src_WT_ and SH2 domain R175K mutant Src unable to bind phosphotyrosines (Src_RK_) (18) were immunoprecipitated from HeLa cells before incubation with MBP-EspJ_EHEC_ and NAD-biotin. EspJ was able to ADP-ribosylate the Src SH2 domain mutant at an efficiency similar to that of WT Src, suggesting that EspJ may select its targets via alternative mechanisms (Fig. 5a and c).

10.1128/mBio.00170-18.3 FIG S2  Kinase targets of EspJ. Proteomic analysis of the kinases enriched after ADP-ribosylation of cell lysate proteins from HeLa (A), A549 (B), Caco2 (C), and Thp1 (D) cells by EspJ. Kinases were selected with a text filter before missing values were replaced by imputing values from a normal distribution and ranking kinases according to the difference in log_2_ LFQ intensity between the presence (+) and absence (−) of EspJ. Imputed values were removed after ranking. The top 20 kinases are displayed for each cell lysate. Log_2_ LFQ intensities are enumerated and represented by a blue-to-red color scale, and missing values are shown in gray. Download FIG S2, PDF file, 0.5 MB. Copyright © 2018 Pollard et al. 2018 Pollard et al. This content is distributed under the terms of the Creative Commons Attribution 4.0 International license.

10.1128/mBio.00170-18.8 TABLE S5  *In vitro* proteomics of EspJ targets. Shown is a spreadsheet of the proteomics-based discovery of EspJ targets from cell lysate including all protein IDs from HeLa, A549, Caco2, and Thp1 cell lysate experiments; the statistical analyses used for volcano plot generation (Fig. 3); and the manually curated lists of identified kinases used in Table 1. Download TABLE S5, XLSX file, 0.9 MB. Copyright © 2018 Pollard et al. 2018 Pollard et al. This content is distributed under the terms of the Creative Commons Attribution 4.0 International license.

### EspJ shows specificity within its NRTK targets.

The proteomics assay highlighted ubiquitous SFKs Src and Yes1, but not Fyn, as EspJ targets. To test this specificity, we assayed the ADP-ribosylation of a panel of immunoprecipitated, GFP-tagged kinases incubated with EspJ and NAD-biotin as described above. EspJ could ADP-ribosylate all of the kinases tested (Src, Yes1, Fyn, Csk, and Abl) (Fig. 5b). Semiquantitative densitometry was used to infer the ADP-ribosylation efficiency from the ratio of ADP-ribosylated protein (anti-biotin antibody Western blot assay) to total protein (anti-GFP antibody Western blot assay) across three to five experiments. This suggested that the SFK Fyn was modified at less than half the efficiency of its family members Src and Yes1 and the non-SFK Abl kinase (Fig. 5C). Interestingly, while Csk was the only kinase to be ADP-ribosylated by EspJ in all four cell lysates (Fig. 3), it was the weakest GFP-tagged substrate in this assay (Fig. 5b and c).

### EspJ inhibits Csk by ADP-ribosylation.

**FIG 6:**
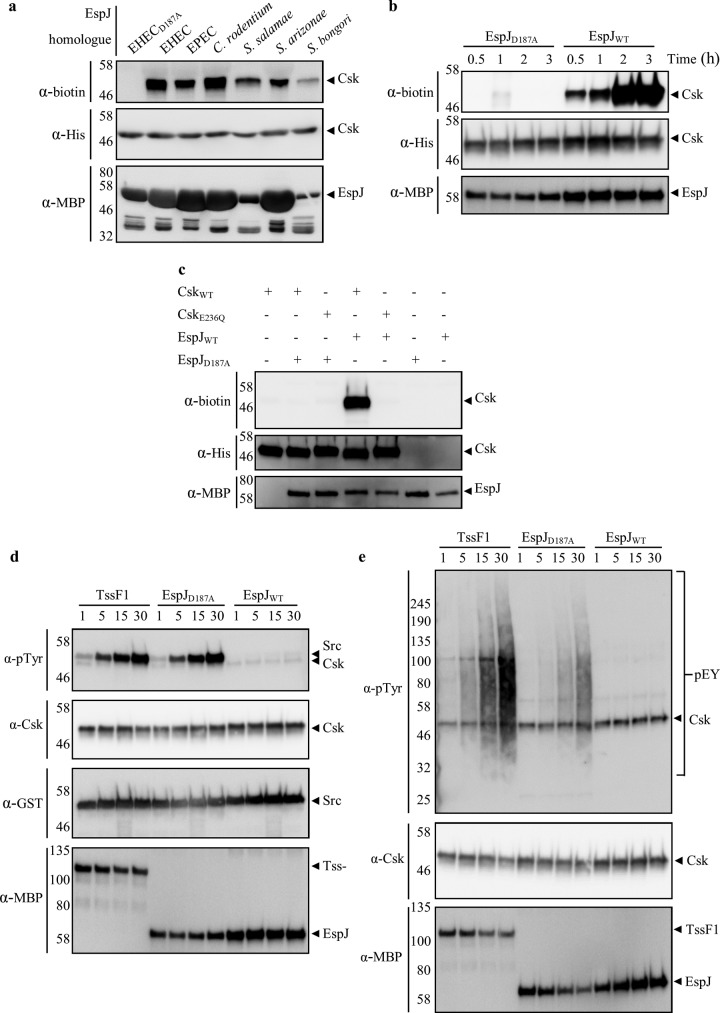
EspJ regulates Csk via inhibitory ADP-ribosylation of E236. MBP-EspJ homologues and catalytic mutants were incubated with His-Csk (WT and mutants) and NAD-biotin and ADP-ribosylated Csk were detected by Western blotting and anti-biotin reagent. (a) WT but not D187A catalytic mutant MBP-EspJ_EHEC_ ADP-ribosylates Csk in a time-dependent manner. (b) EspJ homologues are able to ADP-ribosylate Csk. (c) The E236Q mutation of Csk prevents ADP-ribosylation by EspJ. (d, e) Csk was incubated with MBP-EspJ_WT_, catalytic mutant MBP-EspJ_D187A_, or the control MBP-TssF1 and NAD for 2 h before GST-Src_250–533 K295M/Y416A/E310A_ (d) or poly(Glu_4_, Tyr) (e) substrate addition during a 30-min time course. Kinase activity was assessed by Western blotting with an anti-phosphotyrosine (α-pTyr) antibody. While Csk was able to phosphorylate Src and poly(Glu4, Tyr) after preincubation with EspJ_D187A_ or MBP-TssF1, ADP-ribosylation by MBP-EspJ_WT_ fully inhibited Csk catalytic activity. The values to the left of the gels are molecular sizes in kilodaltons.

As EspJ can target both the SFKs and their inhibitor Csk, we further investigated the EspJ-Csk relationship to understand the interplay among EspJ, SFKs, and Csk. First, as we have previously shown that the EspJ homologues SeoC and SboC can ADP-ribosylate Src, we confirmed their ability to ADP-ribosylate Csk (Fig. 6a). Moreover, while the catalytic mutant (D187A) EspJ_EHEC_ protein was unable to ADP-ribosylate Csk by using biotin-NAD, WT EspJ_EHEC_ modified Csk in a time-dependent manner (Fig. 6b). Finally, mutation of Csk E236, which is analogous to Src E310, prevented ADP-ribosylation, indicating that EspJ_EHEC_ targets the same residue in Src and Csk (Fig. 6c).

As the differential effect of Csk activation or inhibition could have drastic impacts on the regulation of Src kinase, we next tested the effect of Csk ADP-ribosylation by EspJ. ADP-ribosylation of Csk with native NAD was performed for 2 h prior to the addition of kinase buffer and either the synthetic tyrosine kinase substrate poly(Glu_4_, Tyr) or Src_SH1-K295M/Y416A/E310A_ (K295M, catalytic mutant; Y416A, nonautophosphorylated; E310A, cannot be ADP-ribosylated by EspJ). Phosphotyrosine Western blotting revealed that while Csk could still phosphorylate Src/poly(Glu_4_, Tyr) after incubation with NAD and D187A mutant EspJ_EHEC_ or TssF1 (an uncharacterized Pseudomonas aeruginosa protein used as an additional control), WT EspJ_EHEC_ completely abolished Csk kinase activity (Fig. 6d and e). This confirms that EspJ can ADP-ribosylate and inhibit both the SFKs and their inhibitor Csk.

## DISCUSSION

Studying the role of T3SS effectors *in vivo* conventionally relies on the elucidation of pathological, immunologic, or cellular phenotypes. However, as the contributions of many individual effectors to disease are subtle, their roles during *in vivo* infection remain largely unknown. This holds true for EspJ, as an *espJ* mutant colonizes mice as efficiently as the parental WT strain. In this paper, we show, for the first time, that comparing the proteomes of IECs extracted from mice infected with WT and *espJ* mutant C. rodentium reveals the *in vivo* processes affected by EspJ. By applying this approach to other effector mutants, we should be able to illuminate the processes they affect at a molecular level, which will shed new light on pathogen-host interactions.

In this study, we show that EspJ can modify a range of kinases and this is suggested to regulate the host immune response *in vivo*. Previously, Src kinase was the only known substrate for EspJ. Here we show that kinases from the Src, Abl, Csk, Tec, and Syk NRTK families are preferentially ADP-ribosylated by EspJ. Further, while predictions from *in vivo* proteomics hinted that EspJ modulates JAK and FAK, these were not ADP-ribosylated in cell lysate. While expression levels may have contributed to these observations, several JAKs and FAKs are ubiquitously expressed and thus were likely available for ADP-ribosylation by EspJ. Furthermore, our data suggest that within the ubiquitously expressed SFKs, EspJ has a preference for Src and Yes1 over Fyn. Thus, EspJ displays an intricate specificity despite ADP-ribosylating a universally conserved catalytic kinase residue, Src E310. The molecular basis of target specificity is currently not known.

The Src, Tec, and Syk NRTK families are crucial to the host immune response to pathogens. This includes roles in phagocytosis and inflammatory responses, both of which were predicted as EspJ-regulated processes *in vivo* (15, 19–22). A multitude of findings link these kinases to the transduction of signaling from Toll-like receptors (TLR), such as the inflammatory response to bacterial lipopolysaccharide after detection by TLR4 (23), and Syk has been linked to signal transduction after pathogen recognition by IECs (21). The new EspJ substrates that we have identified were either previously observed in IECs or present in the IEC proteome from this study. As IECs are emerging as nonprofessional immune cells critical to the defense against pathogens (24, 25), their inhibition is likely central to the suppression of the host immune response by EspJ *in vivo*.

Our study showed that EspJ can also ADP-ribosylate Csk at the conserved catalytic glutamic acid, ablating its kinase activity. The EspJ homologues SeoC and SboC were also able to ADP-ribosylate Csk, suggesting that the broad regulation of NRTKs may be a conserved strategy employed by the A/E pathogens and *Salmonella*. As Csk is only recruited to SFKs at the membrane upon their activation (26), by inhibiting SFKs, EspJ could potentially prevent the recruitment of Csk. If EspJ did interact with Src and Csk simultaneously, it has the potential to dynamically regulate both proteins, which may be necessary for subtle control of processes such as the dynamics of the EPEC actin pedestals. EspJ inhibition of Csk may also impact other roles for Csk, including the regulation of G proteins or the phosphatases SHP-1 and CD45 (27–29), though further studies are required to understand the global effect of EspJ-Csk interactions. Importantly, while Csk was found to be an EspJ target in lysates of all of the cell line tested, it was modified with less efficiency than the other NRTKs following protein purification. Whether this reduced efficiency is caused by the GFP tag or represents a physiological difference has yet to be determined.

Manipulation of tyrosine kinase signaling is rife during bacterial infections. Tyrosine phosphorylation of EPEC Tir is mediated by Abl and SFKs. Depending on the combination of phosphorylation, either Nck or the phosphatases SHP-1 and -2 may be recruited, stimulating downstream pedestal formation (30, 31) or inhibiting the production of proinflammatory cytokines, respectively (32). Phosphorylation of Helicobacter pylori CagA by Src and Abl results in drastic cell elongation and increased motility (33). *Shigella* recruits Src and Abl to induce the cytoskeletal dynamics necessary for invasion (34), and Btk is required for Shigella flexneri actin tail formation and motility (35). Contrastingly, bacteria may use phosphatases to inhibit NRTKs. *Salmonella* SptP is able to dephosphorylate Syk, (36), and *Yersinia* YopH can dephosphorylate multiple proteins, including NRTKs and targets thereof, controlling internalization, integrins, and immunoreceptor signaling (37).

While EspJ is the only bacterial ART that targets mammalian kinases, other T3SS ARTs can suppress the host immune response, such as HopF2 from the plant pathogen Pseudomonas syringae, which ADP-ribosylates mitogen-activated protein kinase kinases. The Pseudomonas aeruginosa ARTs ExoS and ExoT can inhibit phagocytosis by ADP-ribosylation of a distinct set of host proteins despite sharing 76% sequence identity (38). The EspJ homologues show sequence identity as low as 57% (13). Accordingly, these ARTs may have broader target repertoires, though deeper proteomics analysis is required to fully elucidate their specificity and downstream functions.

## MATERIALS AND METHODS

### Animals.

Animal experiments were performed in accordance with the Animals Scientific Procedures Act 1986 and approved by the local Ethical Review Committee and UK Home Office guidelines. Experiments were designed in agreement with the ARRIVE guidelines (39) for the reporting and execution of animal experiments, including sample randomization and blinding. Pathogen-free female C57BL/6 mice (18 to 20 g, six per group; Charles River, Inc., United Kingdom) were housed in HEPA-filtered cages with sterile bedding (processed corncobs grade 6) and nesting (LBS Serving Technology) and free access to sterilized food (LBS Serving Technology) and water.

### DNA manipulation: plasmid construction and site-directed mutagenesis.

pCB6-GFP plasmids containing Src (WT and R175K mutant), Yes1, Fyn, and Abl were kindly provided by Michael Way (Francis Crick Institute, London). Csk was subcloned into pCB6 and pET28 by restriction enzyme digestion, ligation, and transformation. Site-directed mutagenesis for EspJ D187A and Csk E236Q was performed by nonoverlapping inverse PCR, followed by product phosphorylation and ligation with T4 DNA ligase. For the primers and restriction enzymes used, see Table S1, and for the plasmids used, see Table S2. All enzymes were from NEB.

### Generation of C. rodentium Δ*espJ* and R79A (Δ*ART*) mutants.

The EspJ flanking regions were amplified by PCR with primers 9/10 and 11/12 before digestion alongside the pSEVA612S vector (Table S1). Vectors were assembled by triple ligation before transformation into E. coli CC118λpir (40).

Triparental conjugation, followed by gene excision with arabinose-inducible I-SceI, was utilized to delete *espJ* from C. rodentium as previously described (14). Clones were screened by PCR for *espJ* deletion with primers 15/16 before PCR product sequencing (Table S1). The same methodology was used to reintegrate *espJ-R79A* into the genome with pSEVA612S-(+)300bp-EspJR79A-(−)300bp generated by site-directed mutagenesis (primers 13/14).

### Eukaryotic cell maintenance.

HeLa, A549, Caco2, and Thp1 cells were maintained at 37°C and 5% CO_2_ (Table 2). For polarization, Caco2 cells were seeded into six-well plates at 1 × 10^5^/well and the medium was exchanged at days 2, 4, 6, and 7 to 14. For differentiation, Thp1 cells were seeded at 2 × 10^7^/10-cm dish in the presence of 0.02% phorbol 12-myristate 13-acetate (PMA). Forty-eight hours later, the medium was changed to medium lacking fetal calf serum (FCS) and PMA.

**TABLE 2:** Reagents and resources used in this study^a^

Reagent or resource	Source or reference	Identifier
Bacterial strains		
C. rodentium		
WT	Frankel lab	1CC169
Δ*ART* (*espJ* R79A) mutant	Frankel lab	ICC1472
E. coli		
CC118λpir	39	NA^c^
CC1047	40	NA
Antibodies		
GST, mouse monoclonal 3G10/1B3	Abcam, Inc.	Catalog no. ab92, RRID^d^ AB_307067
MBP, mouse monoclonal MBP-17 (HRP)^b^	Abcam, Inc.	Catalog no. ab49923, RRID AB_881602
His, mouse monoclonal (HRP)	Sigma-Aldrich	Catalog no. A7058, RRID AB_258326
Phosphotyrosine, mouse monoclonal PY20	Sigma-Aldrich	Catalog no. P4110, RRID AB_477342
Csk, rabbit monoclonal C74C1	Cell signaling technology	Catalog no. 4980S, RRID AB_2276592
GFP, mouse monoclonal 9F9.F9	Abcam, Inc.	Catalog no. ab1218, RRID AB_298911
GFP, rabbit monoclonal	Abcam, Inc.	Catalog no. ab290, RRID AB_303395
Peroxidase-AffiniPure goat anti-mouse IgG, Fc gamma fragment specific	Jackson ImmunoResearch, Inc.	Catalog no. 115-035-008, RRID AB_2313585
Peroxidase-AffiniPure goat anti-rabbit IgG, Fc fragment specific	Jackson ImmunoResearch, Inc.	Catalog no. 111-035-008, RRID AB_2337937
Intimin B purified chicken antibody IgY	John Morris Fairbrother; 41	NA
Ki-67 rabbit monoclonal antibody (clone SP6)	Thermo Fisher Scientific	Catalog no. RM-9106-F0; RRID AB_721371
Cy3 AffiniPure goat anti-chicken IgY (IgG) (H+L) J	Jackson ImmunoResearch	Catalog no. 103-005-155; RRID AB_2337379
Alexa Fluor 488 AffiniPure donkey anti-rabbit IgG (H+L)	Jackson ImmunoResearch	Catalog no. 711-545-152; RRID AB_2313584
Chemicals and reagents		
eNAD^+^	Jena Bioscience	Catalog no. CLK 043
Lipofectamine 2000 transfection reagent	Invitrogen	Catalog no. 12566014
AzRB	17	NA
DAPI	Thermo Fisher Scientific	Catalog no. D3571, RRID AB_2307445
Pathogen-free female C57BL/6 mice	Charles River, Inc. (United Kingdom)	Strain code 027

a Shown are the key strains, antibodies, chemicals, and animals used in this study, accompanied by their sources and identifiers.

b HRP, horseradish peroxidase.

c NA, not applicable.

d RRID, research resource identifier.

### IP.

HeLa cells were seeded into six-well plates at 1.5 × 10^5^/well and transfected 24 h later with 2.5 µg of plasmid DNA and 5 µl of Lipofectamine 2000 (Thermo Fisher Scientific) per well. Fourteen hour later, cells were washed twice with phosphate-buffered saline (PBS) and lysed with kinase immunoprecipitation (IP) buffer on ice before sonication. Lysates were clarified at a relative centrifugal force (RCF) of 20,000 for 10 min and precleared for 15 min with Dynabeads protein G (Thermo Fisher Scientific). Incubations were performed at 4°C with rotation. Supernatants were incubated with rabbit anti-GFP antibody 9F9.F9 (Abcam, Inc.)-conjugated Dynabeads for 1 h. Beads were sequentially washed with PBS–0.5% Triton X-100, PBS–0.05% Tween 20, 150 mM NaCl–20 mM Tris (pH 8), and kinase IP buffer before ADP-ribosylation by recombinant MBP-EspH_EHEC_.

### *In vitro* enzyme reactions.

Four micrograms of Csk was added to 4 μg of purified MBP-EspJ_EHEC_ or to EspJ homologues bound to 50 μl of amylose resin. Immunoprecipitated tyrosine kinases were incubated with 2 μg of purified MBP-EspJ_EHEC_. The above ADP-ribosylation reactions with 6-biotin-17-NAD^+^ (NAD-biotin; AMSBIO) were performed for 1 h at room temperature in ADP-ribosylation buffer (Table 3) and terminated with Laemmli buffer and boiling.

**TABLE 3:** Composition of the buffers and cell culture media used in this study

Buffer or medium	Composition
Kinase IP	20 mM Tris (pH 7.5), 150 mM NaCl, 1 mM EDTA, 1 mM EGTA, 1% Triton X-100, 2.5 mM sodium pyrophosphate, 1 mM β-glycerophosphate, 1 mM Na_3_VO_4_, 1 mM dithiothreitol (DTT), 0.5% NP-40, 2 cOmplete mini EDTA-free protease inhibitor tablets (Roche), 5 μl of Benzonase nuclease/10 ml
MBP lysis/gel filtration	500 mM NaCl, 50 mM CAPS,^a^ 5 mM DTT, 10% glycerol (pH 10)
GST lysis/gel filtration	300 mM NaCl, 50 mM Na_2_HPO_4_, 5 mM DTT, 5% glycerol (pH 7.4)
His lysis/gel filtration	50 mM Tris (pH 8), 150 mM NaCl, 10% glycerol, 2 mM DTT
ADP-ribosylation	50 mM Tris (pH 7.4), 1 mM DTT, 60 μM ATP, 5 mM MgCl_2_
1× Kinase buffer	100 mM KCl, 100 mM MOPS,^b^ 10 mM DTT, 10 mM MgCl_2_, 100 µM ATP
Enterocyte dissociation buffer	1× Hanks’ balanced salt solution without Mg and Ca plus 10 mM HEPES, 1 mM EDTA, and 5 µl/ml 2-β-mercaptoethanol
HeLa, A549, or Caco2 cell maintenance	Dulbecco’s modified Eagle’s medium (Sigma-Aldrich) containing 1,000, 1,000, or 4,500 mg/liter glucose, 1% (vol/vol) GlutaMAX (Life Technologies, Inc.), and 10% (HeLa, A549) or 15% heat-inactivated FCS
Thp1 cell maintenance	RPMI supplemented with 10% FCS

a CAPS, *N*-cyclohexyl-3-aminopropanesulfonic acid.

b MOPS, morpholinepropanesulfonic acid.

For testing of Csk inhibition, 1 mM MBP-EspJ_WT_, -EspJ_D187A_, or -TssF1 was incubated with 10 µM His-Csk in ADP-ribosylation buffer. After 2 h, glutathione *S*-transferase (GST)-Src_250–533 K295M/Y416A/E310A_ or poly(Glu_4_, Tyr) (Sigma) was added to 1 mM with 10× kinase reaction buffer and the mixture was incubated for 1, 5, 15, or 30 min.

For proteomics screening of EspJ targets, 400 μg of HeLa, A549, Caco2, or Thp1 cell lysate in ADP-ribosylation buffer was incubated with 20 μg of MBP-EspJ_EHEC_ and eNAD (Jena Biosciences CLK-043) for 3 h at room temperature.

### Click chemistry and pulldown of ADP-ribosylated proteins for LFQ liquid chromatography (LC)-MS.

Cell lysate reaction products were chloroform-methanol precipitated with 4 volumes of methanol, 1.5 volumes of chloroform, and 2 volumes of water before being washed twice with ice-cold methanol and resuspended in 25 μl of 2% SDS–PBS by vortex mixing for 30 min and dilution to 0.5% SDS–PBS with PBS. Biotin was conjugated to proteins by using reaction mixtures containing 100 μM AzRB (azide [Az], arginine [R], and biotin [B]; structure 3 in Fig. 1 of reference 17), 1 mM CuSO_4_, 1 mM tris(2-carboxymethylphosphine) (TCEP), and 100 μM tris(1-benzyl-1*H*-1,2,3-triazol-4-yl)methylamine (TBTA) for 2 h. Reactions were quenched by chloroform-methanol precipitation, and reaction products were resuspended in 50 μl of 2% SDS–PBS as described above.

Samples were diluted with PBS to 0.2% SDS–PBS and incubated with 30 μl of slurry or prewashed NeutrAvidin resin (Thermo Fisher Scientific) per sample for 1 h. Resin was washed three times with 1% SDS–PBS, twice with 4 M urea–PBS, and three times with 50 mM ammonium bicarbonate–H_2_O (pH 8) before digestion with 0.4 μg of trypsin in 50 μl of 50 mM ammonium bicarbonate (pH 8) overnight at 37°C with vortex mixing.

Digested peptide supernatants were combined with supernatants from subsequent washes with 80 μl of 50 mM ammonium bicarbonate and 80 μl of 0.1% trifluoroacetic acid (TFA). Peptides were loaded onto methanol-activated and pre-equilibrated home-made StageTips consisting of three layers of SDB-XC (Empore) solid-phase extraction medium, desalted with H_2_O, and eluted with 79% acetonitrile (ACN) before being vacuum dried and stored at −80°C. For analysis, samples were resuspended in 15 µl of 0.5% TFA–2% ACN and transferred to LC-MS vials.

### LFQ MS—*in vitro* ADP-ribosylation. (i) MS.

For a detailed description of MS and data processing, see Text S1 in the supplemental material. Analysis was performed with an EASY-Spray LC column coupled to a Q Exactive mass spectrometer via an EASY-Spray Source (Thermo Fischer Scientific) in a data-dependent acquisition mode (data were processed using MaxQuant version 1.5.7.4).

10.1128/mBio.00170-18.1 TEXT S1  Supplemental materials and methods used in this study. Details of routine procedures such as bacterial strain growth, protein overexpression and purification, and Western blotting are shown, along with an in-depth description of MS procedures for LFQ LC-tandem MS and TMT-labeled IEC proteome LC-electrospray ionization-tandem MS. Download TEXT S1, DOCX file, 0.02 MB. Copyright © 2018 Pollard et al. 2018 Pollard et al. This content is distributed under the terms of the Creative Commons Attribution 4.0 International license.

### (ii) Volcano plot generation.

Protein groups were analyzed with Perseus (version 1.5.6.0). Potential contaminants, “reverse,” “identified by site,” and proteins with only one unique peptide were removed and data were logarithmized (log_2_). Replicates were grouped and filtered for proteins with at least three valid values across three (Caco2, THP-1) or four (A549, HeLa) replicates in one group. Missing values were imputed with a downshifted normal distribution (1.8 downshift, 0.3 width) for each sample to allow statistical analysis by a two-sided *t* test within the volcano plot function (permutation-based FDR-corrected *P* value of <0.01, *s*_0_ value of 1) (Table S5).

### Infection of mice with C. rodentium*.*

Oral gavage was used to inoculate mice with 200 µl of PBS (uninfected) or C. rodentium (ca. 5 × 10^9^ CFU). The inoculum was determined by counting CFU after plating on LB agar supplemented with nalidixic acid, and colonization was monitored by counting CFU per gram of stool sample on days 3, 6, 7, and 8.

### Tissue staining and measurement.

A 0.5-cm section of the distal colon was washed with PBS, fixed in 1 ml of buffered formalin, paraffin embedded, and sectioned at 5 µm. Sections were stained with hematoxylin and eosin (H&E) or treated with sodium citrate antigen demasking solution prior to immunofluorescence staining (antibodies, Table 2). CCH was assessed by measuring at least 20 crypt lengths in samples from at least four mice per group.

### Enterocyte extraction.

Eight days postinfection, a 4-cm section of the terminal colon was cut lengthwise and incubated for 45 min at 37°C in 4 ml of enterocyte dissociation buffer. Enterocytes were harvested by centrifugation at an RCF of 2,000, washed twice in PBS, and stored at −80°C prior to labeling.

### Digestion and TMT labeling.

Enterocytes were dissolved in 100 µl of 0.1 M triethylammonium bicarbonate (TEAB)–0.1% SDS and lysed by pulse probe sonication. Eighty micrograms of protein per sample was SpeedVac dried and then resuspended in 100 µl of 4% SDS–100 mM TEAB–15 mM TCEP, assisted with an ultrasonic bath. After reduction at 56°C for 20 min, samples were cooled to 25°C and alkylated with iodoacetamide (IAA) for 30 min. Proteins were purified by 20% trichloroacetic acid precipitation, followed by one wash with ice-cold acetone before resuspension in 100 mM TEAB and digestion with 3 µg of trypsin (MS grade; Pierce) at 37°C for 18 h. Forty micrograms of protein digest was labeled with 0.4 mg of TMT10plex as instructed by the manufacturer (Thermo Fisher Scientific).

### IEC proteome MS. (i) MS.

For a detailed description of MS and data processing, see Text S1 in the supplemental material. Briefly, samples were fractionated with a U3000 high-performance liquid chromatography system (Thermo Fisher Scientific) coupled to an XBridge ethylene-bridged hybrid C_18_ column (Waters). Peptides were injected onto the Orbitrap Fusion Tribrid mass spectrometer coupled to a U3000 RSLCnano ultrahigh-performance liquid chromatography system (Thermo Fisher Scientific) loading onto a PepMap C_18_ trap and separated on a PepMap C_18_ column. Data acquisition was done by the SPS10-MS3 method.

### (ii) Bioinformatics analysis.

Protein groups were processed with Perseus (version 1.5.6.0). Absolute intensities were logarithmized (log_2_), and those with only one unique peptide were removed. Ratios between conditions were created, and proteins with a fold change of >1.5 were analyzed further. Venn diagrams were created in BioVenn (41), and heat maps were created in Perseus.

GO terms were analyzed with the ClueGO Cytoscape plug-in (version 3.6.0) (42, 43). The minimum and maximum GO levels were set to 3 and 7, respectively. GO terms with a *P* value of <0.01 were selected with a minimum of five proteins and 5% of the GO term proteins. GO term grouping and fusion were utilized, and the most significant (Bonferroni-corrected *P* value) GO term was used for visual representation.

The kinase prediction function (KEA) of X2K (16) was used in isolation to predict upstream regulatory kinases from the group of 457 proteins upregulated in the Δ*ART* versus WT samples or from 10 randomly generated sets of 457 proteins from the total of 7,400 proteins identified.

### Availability of data.

The data sets obtained in this study can be found in PRIDE project PXD008533.
