# Supplementary figures and images for: Broad-Spectrum Regulation of Nonreceptor Tyrosine Kinases by the Bacterial ADP-Ribosyltransferase EspJ
Source: mBio. 2018 Apr 10;9(2):e00170-18. doi: 10.1128/mBio.00170-18 (PMC5893879; doi:10.1128/mBio.00170-18)

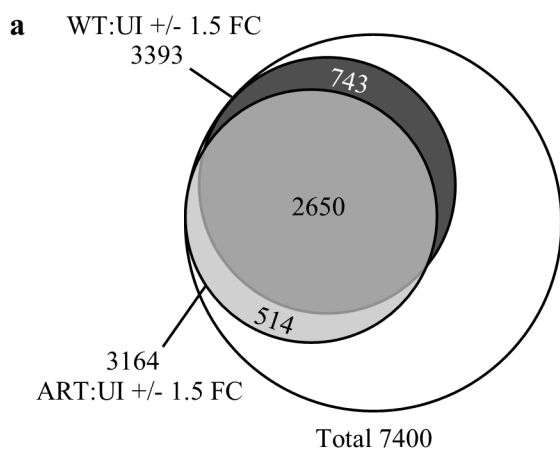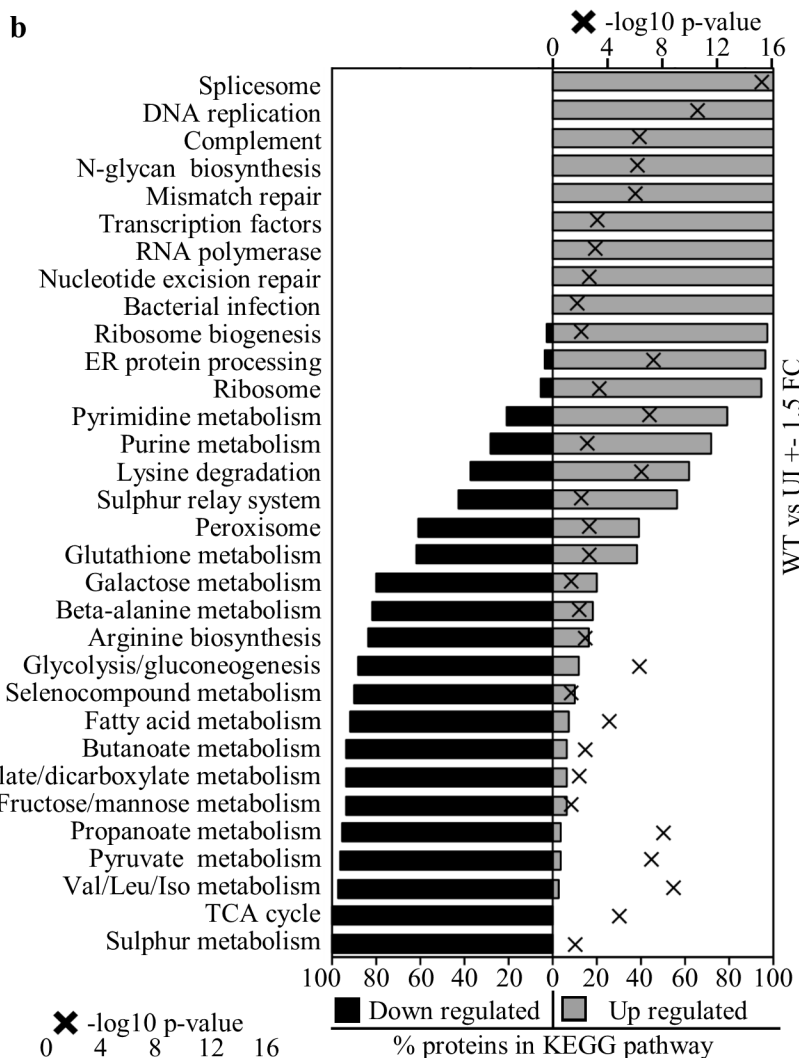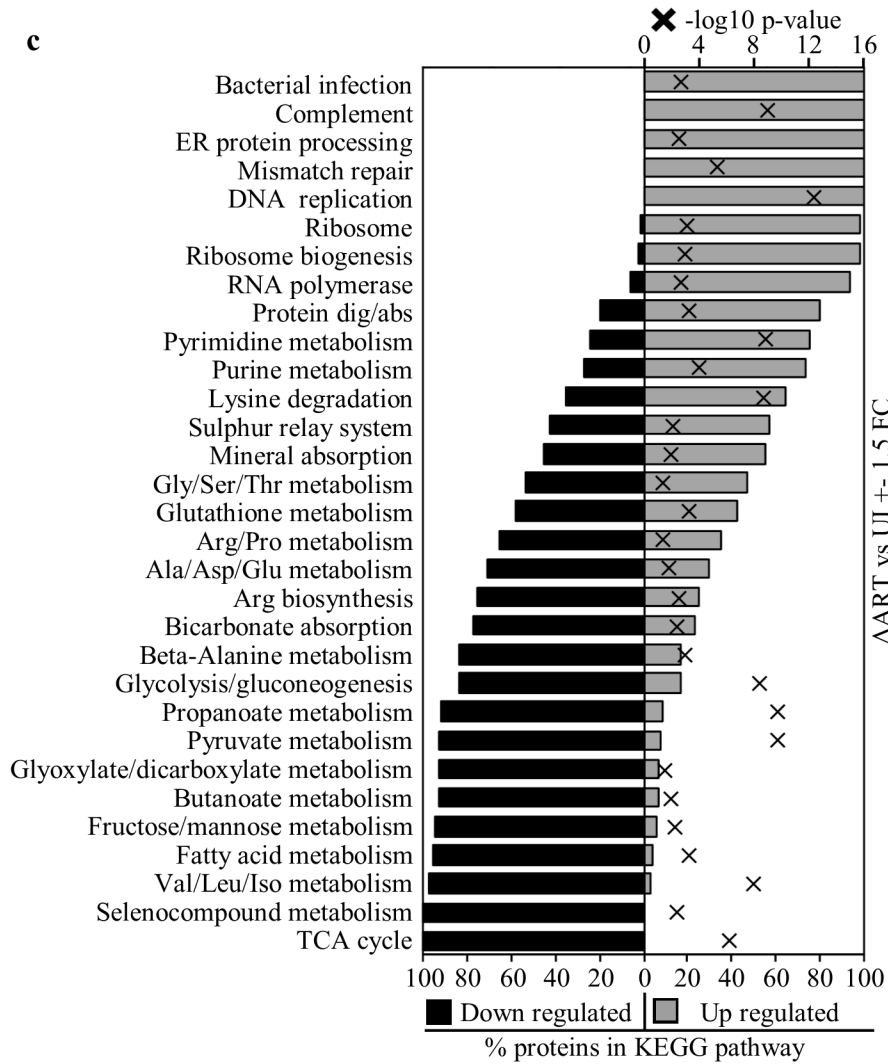

Supplement: FIG S1 [file mbo002183816sf1.pdf]

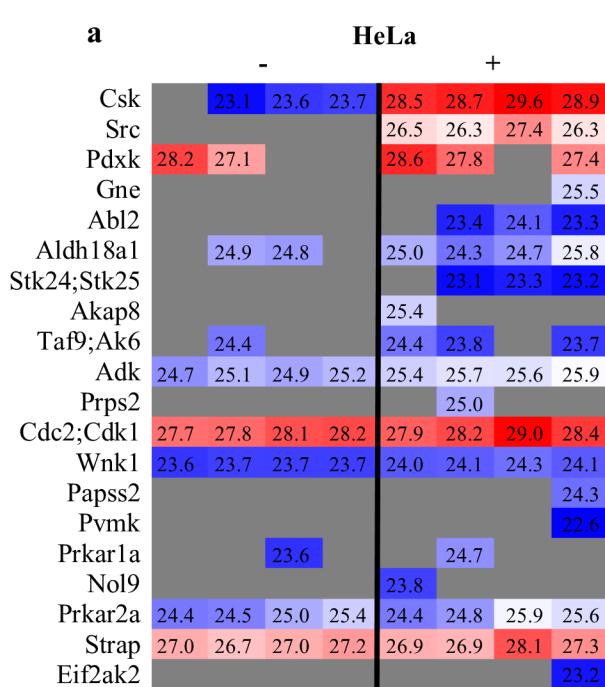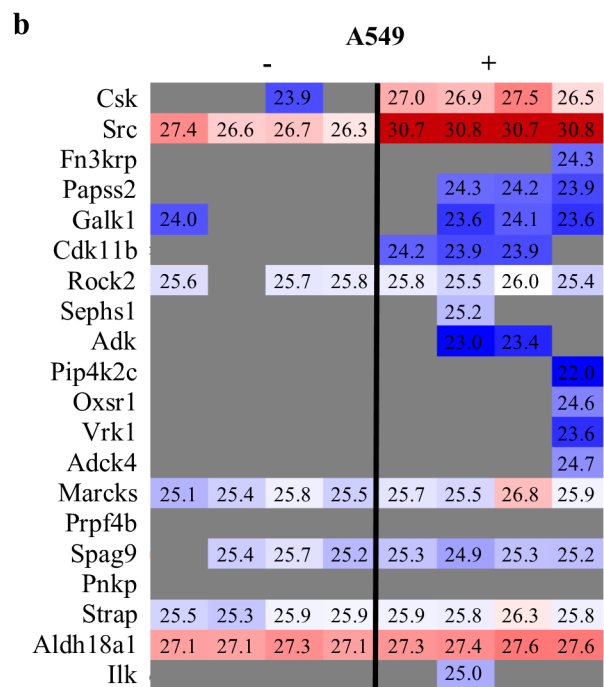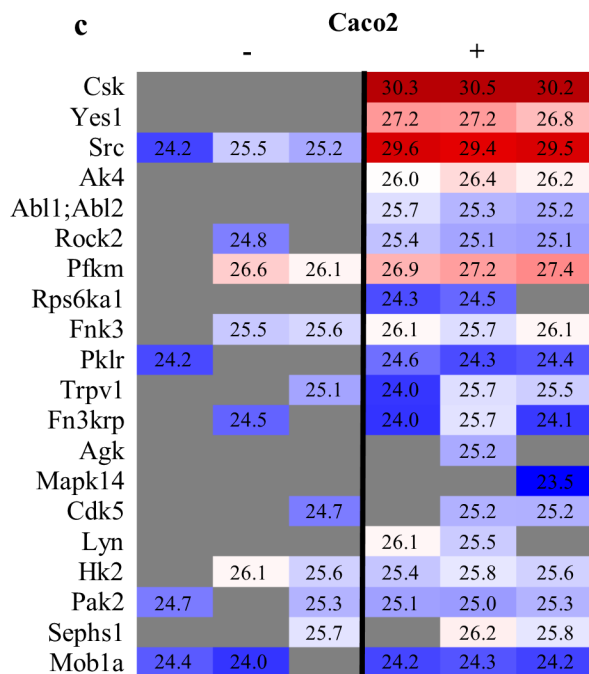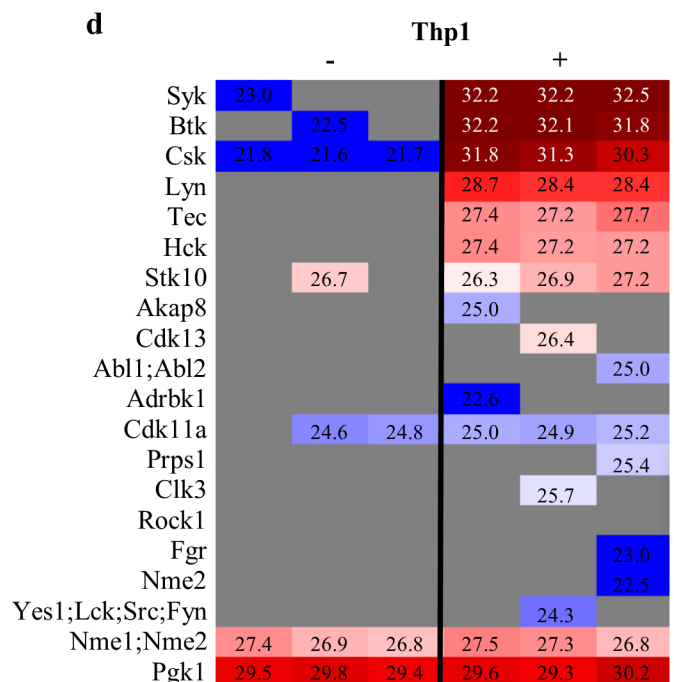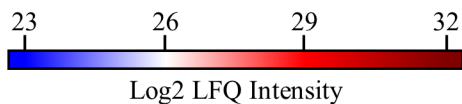

Supplement: FIG S2 [file mbo002183816sf2.pdf]
