# Supplementary material for: Broad-Spectrum Regulation of Nonreceptor Tyrosine Kinases by the Bacterial ADP-Ribosyltransferase EspJ
Source: mBio. 2018 Apr 10;9(2):e00170-18. doi: 10.1128/mBio.00170-18 (PMC5893879; doi:10.1128/mBio.00170-18)
Supplement: TABLE S1 [file mbo002183816st1.docx]

**Table S1: List of primers**

| # | **Plasmid/Use** | **Primer Sequence (5’-3’) (Restriction Sites Underlined)** | **Restriction site** |
| --- | --- | --- | --- |
| 1  2 | pMALXE-EHEC-EspJ_D187A_ | F-GGAGCAAAAGTATATCCCGCTACATCATGCTCTCTGAGAC  R- GTCTCAGAGAGCATGATGTAGCGGGATATACTTTTGCTCC | -  - |
| 3  4 | pCB6-Csk | F- TTCAGATCTGGTACCATGTCAGCAATACAGGCCGC  R- ATAAGAAGCGGCCGCGCCGCCGCCCAGGTGCAGCTCGTGGGT | BglII  NotI |
| 5  6 | pET28-Csk-WT | F- GGGAATTTCCATATGTCAGCAATACAGGCCGCCT  R- CACGGATCCTCACAGGTGCAGCTCGTGGG | NdeI  BamHI |
| 7  8 | pET28-Csk-E236Q | F- CAAGCCTCAGTCATGACGCAAC  R- AGCCAGGAAGGCCTGGG | -  - |
| 9  10 | EspJ -300 bp fragment | F- CTAGTCTAGAGCAAAATAAATATATAGAAGTTATGAATG  R- GGAATTCCATATGGATAAACTCCTTGTCGCCATATT | XbaI  NdeI |
| 11  12 | EspJ +300 bp fragment | F- GGAATTCCATATGTCTTAAATATACTTAGATATAATAAGGTG  R- CTGGAGCTCGAGCAACACATCCGGGATC | NdeI  SacI |
| 13  14 | *C. rodentium* EspJ R79A | F- GCAGTTCAAGACAATCAATTCACTGAT  R- TACAGCAACGAAATTGCGTTTTATA | -  - |
| 15  16 | PCR screen *C. rodentium* mutants EspJ +/-350bp | F- GTTGCTTTGTTCTCTGCCAC  R- GGCGTTTTTTGTAAACGGAGC | -  - |
