# Supplementary material for: Broad-Spectrum Regulation of Nonreceptor Tyrosine Kinases by the Bacterial ADP-Ribosyltransferase EspJ
Source: mBio. 2018 Apr 10;9(2):e00170-18. doi: 10.1128/mBio.00170-18 (PMC5893879; doi:10.1128/mBio.00170-18)
Supplement: TABLE S2 [file mbo002183816st2.docx]

**Table S2: List of plasmids**

| **Plasmid** | **Description** | **Source / Reference** |
| --- | --- | --- |
| pMALXE | Expression MBP tagged fusion proteins in *E. coli* expression host | (Moon et al) |
| pICC2446 | pMALXE- expression of MBP tagged EPEC EspJ | Pollard *et al* 2016 |
| pICC2447 | pMALXE—expression of MBP tagged EHEC EspJ | Pollard *et al* 2016 |
| pICC2448 | pMALXE- expression of MBP tagged *C.rodentium* EspJ | Pollard *et al* 2016 |
| pICC2449 | pMALXE - expression of MBP tagged *S.salamae* SeoC | Pollard *et al* 2016 |
| pICC2450 | pMALXE - expression of MBP tagged *S.arizonae* SeoC | Pollard *et al* 2016 |
| pICC2451 | pMALXE - expression of MBP tagged *S.bongori* SboC | Pollard *et al* 2016 |
| pICC2553 | pMALXE – expression of MBP-EHEC EspJ D187A | This study |
| pGEX-KG | Expression GST tagged fusion proteins in *E. coli* expression  host | GE Healthcare |
| pICC2486 | pGEX-KG– expression of GST tagged Src_250-533-K295M/Y416F/E310A_ | Pollard *et al* 2016 |
| pET28a | Expression of N‑terminally hexahistidine tagged proteins in *E. coli* expression host | GE Healthcare |
| pICC2550 | pET28a – expression of His tagged Csk | This study |
| pICC2551 | pET28a – expression of His tagged Csk E236Q | This study |
| pCB6-GFP | Expression of GFP tagged proteins in mammalian cells | Newsome *et al* 2006 |
| pICC2286 | pCB6- expression of GFP tagged Src | “ |
| pICC2347 | pCB6- expression of GFP tagged Src R175K | “ |
| pICC2287 | pCB6- expression of GFP tagged Yes1 | “ |
| pICC2285 | pCB6- expression of GFP tagged Fyn | “ |
| pICC2288 | pCB6- expression of GFP tagged Abl | “ |
| pICC2549 | pCB6- expression of GFP tagged Csk | This study |
| pSEVA612S | Plasmid for mutation of EspJ in *C. rodentium* | Herrero 1990 |
| pICC2562 | pSEVA612S – (+)300bp-(-)EspJ-(-)300bp - no espJ | This study |
| pICC2561 | pSEVA612S – (+)300bp-EspJ_R79A_-(-)300bp | This study |
| pACBSR | Endonuclease I-SceI under arabinose induction | Ruano-Gallego 2015 |
| pRK2013 | For mobilization of non-self-transmissible plasmids | Figurski 1979 |
