# Supplementary material for: Broad-Spectrum Regulation of Nonreceptor Tyrosine Kinases by the Bacterial ADP-Ribosyltransferase EspJ
Source: mBio. 2018 Apr 10;9(2):e00170-18. doi: 10.1128/mBio.00170-18 (PMC5893879; doi:10.1128/mBio.00170-18)
Supplement: TEXT S1 [file mbo002183816s1.docx]

**Supplementary information**

**Bacterial strain growth and manipulation**

Bacteria were routinely cultured in Luria‑Bertani (LB) broth at 37°C with ampicillin (100 μg/ml), gentamycin (10 μg/ml), kanamycin (50 μg/ml), streptomycin (50 μg/ml), or nalidixic acid (50 μg/ml).

**Protein overexpression and purification**

EspJ/Src/Csk were expressed in BL21 STAR *E. coli.* Stationary phase cultures were sub‑cultured 1:100 in 1 L of LB. Expression was induced at OD_600nm_ of 0.6-1.0 with 0.5 mM IPTG, at 18 °C overnight. Cells were harvested at 4500 RCF and resuspended in 40 ml of MBP lysis buffer, GST lysis buffer or His lysis buffer (table x), lysed by sonication and clarified at 40000 RCF.

GST-Src/His-Csk were purified using GSTrap 4B/HiTRAP Talon sepharose columns (GE Healthcare), MBP‑EspJ was purified by amylose resin (NEB) according to manufacturer’s instructions. Proteins were further purified using a Superdex S200 10/300 gel filtration column.

**Western immunoblotting**

Proteins were separated by SDS‑PAGE with 4-20% TGX gels (Biorad) and transferred to PVDF membranes using the Biorad Trans‑Blot^®^ semi‑dry transfer cell. Membranes were blocked for 1 h with 3% BSA/PBST (Streptavidin‑HRP), or 5% skim milk/PBST (all other antibodies) before sequential incubation with primary and secondary antibodies in 3% BSA/PBST or 1% skim milk/PBST for 1 h each with washing in between (table x). Blots were visualised using EZ-ECL (biological industries) and a Fuji LAS3000 imager.

**Label free mass spectrometry**

Analysis used an EASY-Spray LC Column of 50 cm by 75 µm inner diameter (Thermo Fischer Scientific), with a 2h acetonitrile gradient in 0.1% aqueous formic acid (FA), at a flow rate of 250 nl/min. Easy nLC‑1000 was coupled to a Q Exactive mass spectrometer via an easy‑spray source (Thermo Fisher Scientific). The Q Exactive was operated in a data‑dependent acquisition mode with survey scans acquired at a resolution of 75,000 at m/z 200 (transient time 256 ms). Up to ten of the most abundant isotope patterns with charge +2 or higher from the survey scan were selected with an isolation window of 3.0 m/z and fragmented by higher-energy collisional dissociation with normalized collision energies of 25. The maximum ion injection times for the survey scan and the MS/MS scans (acquired with a resolution of 17,500 at m/z 200) were 20 and 120 ms, respectively. The ion target value for MS was set to 10^6^ and for MS/MS to 10^5^, and the intensity threshold was set to 8.3 × 10^2^.

*Data processing*

Data were processed using MaxQuant (45) (version 1.5.7.4) and peptides identified by matching MS/MS spectra against the human proteome (Uniprot) using Andromeda search engine (46). No fixed modifications were set. Methionine oxidation and N‑terminal acetylation were set as variable modifications. Reference proteomes were digested *in silico* using the trypsin/P setting with up to three missed cleavages. Standard settings for label free quantification were selected, the false discovery rate set to 0.01, and peptides matched between runs. Other parameters were used as pre‑set in MaxQuant. Datasets can be found as PRIDE project [PXD008533](https://www.ebi.ac.uk/pride/archive/projects/PXD008533).

**IEC proteome mass spectrometry**

*Basic reverse‑phase peptide fractionation*

Ten samples were mixed and SpeedVac dried, before fractionation on a U3000 HPLC system (Thermo Fisher) with an XBridge BEH C18 column (2.1 mm id x 15 cm, 130 Å, 3.5 µm, Waters) at pH 10, flow rate 200 µl/min in 30 min linear gradient from 5 - 35% acetonitrile /NH_4_OH. Fractions were collected every 30 sec into a 96-wellplate then concatenated (equally by retention time interval to 24 fractions) and SpeedVac dried.

*LC-ESI-MS/MS analysis*

Peptides were resuspended in 0.5% FA and 1/3 were injected on the Orbitrap Fusion Tribrid mass spectrometer coupled to U3000 RSLCnano UHPLC system (Thermo Fisher). Peptides were first loaded to a PepMap C18 trap (100 µm i.d. x 20 mm, 100 Å, 5 µm) for 10 min at 10 µl/min with 0.1% FA/H_2_O, then separated on a PepMap C18 column (75 µm i.d. x 500 mm, 100 Å, 2 µm) at 300 nl/min and a linear gradient of 4-33.6% ACN/0.1% FA in 120 min /cycle at 150 min per fraction. The data acquisition used the SPS10-MS3 method with Top Speed at 3s per cycle time. The full MS scans (m/z 380-1500) were acquired at 120,000 resolution at m/z 200 with a lock mass at 445.12003, and the AGC was set at 4e5 with 50 ms maximum injection time. The most abundant multiply-charge ions (z = 2-6, above 5000 counts) were subjected to MS/MS fragmentation by CID (35% CE) and detected in ion trap for peptide identification. The isolation window by quadrupole was set m/z 1.0, and AGC at 1e4 with 35 ms maximum injection time. The dynamic exclusion window was set ±7 ppm with a duration at 60 sec, and only single charge status per precursor was fragmented. Following each MS2, the 10-notch MS3 was performed on the top 10 most abundant fragments isolated by Synchronous Precursor Selection (SPS). The precursors were fragmented by HCD at 60% CE then detected in Orbitrap at m/z 110-400 with 50K resolution for peptide quantification data. The AGC was set 1e5 with maximum injection time at 86 ms.

*Database searching and quantification*

The LC-MS/MS data were processed in Proteome Discoverer 2.1 (Thermo Fisher Scientific) using the SequestHT search engine to search against a combined protein database comprised of the mouse protein database (17,555 entries, Swiss-prot, December 2015), a *Citrobacter rodentium* (5020 entries, Uniprot, December 2015) and the in-house common contaminate database. The precursor mass tolerance was set at 30 ppm and the fragment ion mass tolerance was set at 0.5 Da. Spectra were searched for fully tryptic peptides with maximum 2 miss-cleavages. Carbamidomethyl (C) and TMT6plex (Peptide N-terminus and K) were set as static modifications, and Deamidation (N, Q) and Oxidation (M) were set as dynamic modifications. Peptides were validated by Percolator using the Decoy database search and only peptides at high confidence were used in the protein identification. The search result was further filtered where the protein FDR was set at 0.01 (strict) and 0.05 (relaxed). The TMT10plex reporter ion quantifier used 30 ppm integration tolerance on the most confident centroid peak at the MS3 level. Reporter abundance used S/N and the co-isolation threshold is set 75%. Both unique and razor peptides were used for quantification. Only master proteins at high or medium FDR were exported for further filtering/processing.
